# Prevalence of low glomerular filtration rate, proteinuria and associated risk factors in North India using Cockcroft-Gault and Modification of Diet in Renal Disease equation: an observational, cross-sectional study

**DOI:** 10.1186/1471-2369-10-4

**Published:** 2009-02-17

**Authors:** Narinder P Singh, Gopal K Ingle, Vinay K Saini, Ajita Jami, Pankaj Beniwal, Madan Lal, Gajender S Meena

**Affiliations:** 1 Department of Internal Medicine, Maulana Azad Medical College, New Delhi, India; 2 Department of Community Medicine, Maulana Azad Medical College, New Delhi, India; 3 Medical Student, Maulana Azad Medical College, New Delhi, India

## Abstract

**Background:**

Chronic kidney disease (CKD) is increasingly being recognized as an emerging public health problem in India. However, community based estimates of low glomerular filtration rate (GFR) and proteinuria are few. Validity of traditional serum creatinine based GFR estimating equations in South Asian subjects is also debatable. We intended to estimate and compare the prevalence of low GFR, proteinuria and associated risk factors in North India using Cockcroft-Gault (CG) and Modification of Diet In Renal Disease (MDRD) equation.

**Methods:**

A community based, cross-sectional study involving multistage random cluster sampling was done in Delhi and its surrounding regions. Adults ≥ 20 years were surveyed. CG and MDRD equations were used to estimate GFR (eGFR). Low GFR was defined as eGFR < 60 ml/min/1.73 m^2^. Proteinuria (≥ 1+) was assessed using visually read dipsticks. Odds ratios, crude and adjusted, were calculated to ascertain associations between renal impairment, proteinuria and risk factors.

**Results:**

The study population had 3,155 males and 2,097 females. The mean age for low eGFR subjects was 54 years. The unstandardized prevalence of low eGFR was 13.3% by CG equation and 4.2% by MDRD equation. The prevalence estimates of MDRD equation were lower across gender and age groups when compared with CG equation estimates. There was a strong correlation but poor agreement between GFR estimates of two equations. The survey population had a 2.25% prevalence of proteinuria. In a multivariate logistic regression analysis; age above 60 years, female gender, low educational status, increased waist circumference, hypertension and diabetes were associated with low eGFR. Similar factors were also associated with proteinuria. Only 3.3% of subjects with renal impairment were aware of their disease.

**Conclusion:**

The prevalence of low eGFR in North India is probably higher than previous estimates. There is a significant difference between GFR estimates derived from CG and MDRD equations. These equations may not be useful in epidemiological research. GFR estimating equations validated for South Asian populations are needed before reliable estimates of CKD prevalence can be obtained. Till then, primary prevention and management targeted at CKD risk factors must play a critical role in controlling rising CKD magnitude. Cost-benefit analysis of targeted screening programs is needed.

## Background

Economic development and changing lifestyle habits are increasingly having a significant impact on the public health scenario of tropical nations. This epidemiological transition has necessitated reconsideration of public health policies. Whilst the focus previously was entirely on communicable diseases, mostly infectious, the increasing prevalence of non-communicable diseases and their risk factors is worrisome. Considering the monetary constraints and the national and global commitments to limit or eradicate infectious diseases, the increasing burden of lifestyle disorders is bound to introduce resource crunching on the health sector of these economies. One such situation being faced now by India and other nations is the increasing prevalence of chronic kidney disease (CKD). India at present has the world's largest population of diabetics [1] and obesity has long been recognized as an emerging epidemic. The CURES cohort suggested that every fifth person in India is hypertensive [2]. Considering the high prevalence of CKD risk factors it has long been presumed that CKD represents a major public health problem in India, at least in urban cities. However, in view of an overburdened health setup and absence of national registries, the true magnitude of CKD has largely been controversial. Only two population-based studies estimating CKD prevalence in India are available [3-5]. But these studies either used crude criteria for defining CKD or did not cover the entire spectrum of population. The issue of obtaining an accurate estimate of CKD prevalence is further limited by lack of glomerular filtration rate (GFR) estimating equations validated for Indian population. Majority of these equations have been derived predominantly from Caucasian populations and may not be reliable and accurate in Indian context [6,7]. Cockcroft-Gault (CG) and Modification of Diet in Renal Disease (MDRD) equations have been used worldwide in epidemiological studies on CKD. However, till date no community-based study has compared GFR estimates derived from these two equations in Indian population.

In light of the limitations of above studies and the amenability of limiting CKD costs with early detection and primary prevention, we conducted this study. We intended to estimate the prevalence of low glomerular filtration rate, proteinuria and CKD associated risk factors in a population based cross-sectional survey in North India.

## Methods

### Study design

The study was conducted in Delhi and adjoining regions. Delhi, being the only metropolitan city in North India, has population representation from almost every state. While majority of Delhi can be classified as urban or periurban, the surrounding regions are primarily rural. Thus we were able to evaluate the entire socioeconomic spectrum of population. The study was designed as a cross-sectional survey and involved multistage random cluster sampling. The study was funded by a government-based organization (UGC; University Grants Commission) and was conducted from April 2005 to December 2007 after approval by the ethical committee of Maulana Azad Medical College.

### Subjects and Sample size

Delhi and its adjoining regions have a population of 13 × 10^6 ^with 55% of population aged 20 years or above. To ensure an adequate representation, the entire population was divided into clusters and sampling was done proportionate to cluster population. Only adults (≥ 20 years) were included. We anticipated the prevalence of low eGFR to be 5–10% based on estimates of recent epidemiological studies from Asian and African countries [8-10]. We intended to estimate prevalence within 1 percentage point (*d *= 0.01) of true value with 95% confidence. Thus the required sample size, for multistage random cluster sampling, was 6,914. In the field, local health workers facilitated the interaction of surveyors with community. Selected patients were invited to visit local health center where detailed evaluation was done.

### Method

A pre-structured and validated questionnaire was administered to all participants who presented for health checkup. The questionnaire collected information about demographics, lifestyle habits and CKD risk factors. Anthropometric parameters were assessed using standardized techniques. Body weight and height were measured in light clothes without footwear. Every participant underwent a blood pressure measurement by two physicians at an interval of 10 minutes and the mean of two measurements was finally recorded. A spot urinary protein was assessed using dipsticks. 5 ml of fasting venous blood sample was taken for assessing biochemical variables. All participants gave informed consent.

Serum creatinine was estimated using modified Jaffe's method on a Hitachi 911 auto analyzer. All samples were analyzed in the same laboratory on same equipment throughout the duration of study. Twice daily quality control checks were done. The upper limit for normal serum creatinine levels was 1.2 mg/dl. We however did not standardize the serum creatinine measurements to those used in Modification of Diet in Renal Disease (MDRD) equation.

### Evaluation criteria

All subjects were assessed for biochemical and clinical variables using established guidelines and norms.

#### Hypertension (HTN)

Hypertension was defined as per JNC-7 guidelines [11]. Systolic blood pressure (SBP) ≥ 140 mmHg and diastolic blood pressure (DBP) ≥ 90 mmHg were used as cutoffs. Patients taking anti-hypertensive drugs were also included, even if they had blood pressure values lower than cutoff. However, no adjustments in cutoff values were made for diabetic status. Also we did not stratify HTN by severity or by anti-hypertensive drug intake.

#### Diabetes mellitus (DM)

Known diabetics on treatment were considered diabetic regardless of their glycemic control. For others, a 12-hour fasting blood sugar level of ≥ 126 mg/dl was used as cutoff [12].

#### Proteinuria

Proteinuria was estimated using visually read dipsticks (Teco diagnostics, USA). None and trace urinary protein were classified as no proteinuria and rest (1+, 2+ and 3+) as proteinuria. Women having menstrual periods were studied remote from their periods. Pregnant women and subjects with active urinary tract infections were excluded.

#### Renal impairment

The glomerular filtration rate (GFR) was estimated using Cockcroft-Gault (CG) formula corrected for sex and body surface area (BSA). Use of BSA corrected CG equation enabled direct comparisons with GFR estimates of Modification of Diet in Renal Disease (MDRD) equation. We preferred to use CG-GFR estimates over CG-creatinine clearance estimates, as CG-GFR equation is better suited to estimate subnormal GFR in Indian population besides being corrected for renal tubular secretion of creatinine [7].

#### CG/BSA formula

(1) Creatinine clearance (ml/min/1.73 m^2^) = [(140-age)*(weight)/(serum creatinine*72*BSA/1.73)]*(0.85 if female)

BSA (m^2^) = 0.20247*(height in meters)^0.725^*(weight in kg)^0.425^

CG-GFR estimate (ml/min/1.73 m^2^) = 0.84* (creatinine clearance by equation 1)

We also used abbreviated four variable Modification of Diet in Renal Disease (MDRD) equation to estimate GFR. Since prevalence estimates of CKD are dependent on the nature of GFR estimating equations, it enabled us to compare prevalence estimates obtained from two equations.

(2) MDRD-GFR (ml/min/1.73 m^2^) = 186*(serum creatinine)^-1.154^*(age)^-0.203^*(0.742 if female)

The estimated GFR (eGFR) was then used to classify subjects into Kidney Disease Outcomes Quality Initiative (K/DOQI) stages of CKD [13]. Renal impairment was defined as eGFR less than 60 ml/min/1.73 m^2^. Thus stage 3, 4 and 5 of KDOQI were grouped as renal impairment.

#### Obesity

Weight (in kilograms) and height (in meters) were used to calculate body mass index (BMI). The classification of BMI, as recommended by WHO, was employed [14]. BMI ≥ 25 kg/m^2 ^was used as the cutoff for obesity [15]. Additionally, waist circumference (WC) was used to assess body fat distribution. WC was measured using smallest circumference between lower ribs and iliac crests. The mean of two measurements was taken as the final value. A WC > 85 cm and > 80 cm was used as the cutoff for men and women respectively [15].

#### Smoking and Alcohol intake

Smoking was dichotomized as current smokers or not. Alcohol intake was dichotomized on basis of intake frequency, at least weekly against monthly or rarely. No attempt was made to quantify amount of smoking (packs per day) or alcohol intake.

### Statistical Analysis

Results were expressed as absolute numbers, proportions, means with standard deviations or medians with range. Nominal variables were analyzed for associations using Chi-square test and crude (unadjusted) odds ratio were calculated where appropriate. Interval variables were analyzed using *t*-test for normally distributed variables and Wilcoxon's rank sum test for non-normal distributions. Multivariate logistic regression models were framed adjusting for all variables and significant two-way interactions between variables. Backward selection was used to drop insignificant terms. All statistical tests were done using SPSS software (SPSS Inc., Chicago, IL, USA; version 15).*p *< 0.05 was taken as significant.

## Results

A total of 5,563 subjects were surveyed over the duration of study. 311 (5.6%) subjects were excluded because they refused to participate. Thus effectively we had 5,252 subjects. 60% of these (3,155) were men and 40% (2,097) women. The desired sample size could not be achieved due to funding constraints. Our study population characteristics were similar to those of census population of Delhi, especially in terms of distribution across age groups and gender (data not shown).

### Prevalence of low glomerular filtration rate

**Figure 1:**
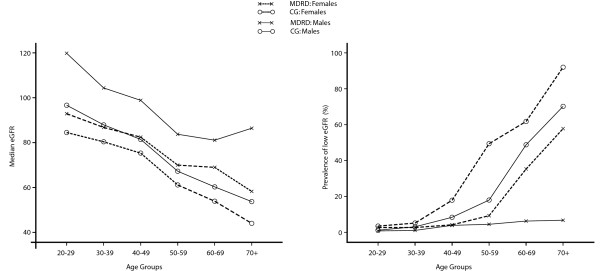
**Median eGFR and prevalence of low eGR across age groups in study population**. The median eGFR progressively decreased across age groups and was lower for females than males. Low eGFR was defined as eGFR < 60 ml/min/1.73 m^2^. Elderly (aged above 60 years) and females had highest prevalence of low eGFR. eGFR, estimated glomerular filtration rate; CG/BSA, Cockcroft-Gault equation corrected for body surface area; MDRD, modification of diet in renal disease equation.

**Figure 2:**
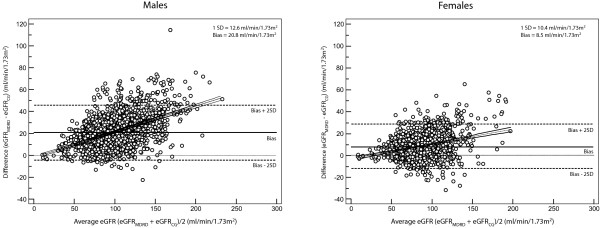
**Bland-Altman plot of the eGFR measurements by CG and MDRD equations**. Differences between eGFR measurements by CG/BSA and MDRD equation were plotted against the mean of two. The solid horizontal line represents the mean difference or the estimated bias. The MDRD equation had a positive bias as compared to CG/BSA equation, suggesting that MDRD equation overestimated GFR in the study population. The limits of agreement between two equations were broad (Males: -4.27 to 45.95 ml/min/1.73 m^2^, Females: -11.88 to 28.82 ml/min/1.73 m^2^). The thin dotted line represents a linear fit of data point. There was an increase in variance of MDRD eGFR estimates across eGFR range. eGFR, estimated glomerular filtration rate; CG/BSA, Cockcroft-Gault equation corrected for body surface area; MDRD, modification of diet in renal disease equation.

K/DOQI guidelines were used for staging estimated GFR (eGFR). Table 1 lists the prevalence of stage 3–5 of eGFR using CG and MDRD equation. The unstandardized prevalence for low eGFR in our study population using CG/BSA equation (Equation 1) was 13.3%. When the population was stratified using gender, the prevalence of low eGFR in males was 11.1% compared to 16.6% of females. MDRD equation (Equation 2) gave lower estimates; overall 4.2%, males 2.7% and females 6.3%. Figure 1 compares the median eGFR and prevalence of low eGFR obtained using the two equations across age groups and gender. There was an increasing prevalence of reduced eGFR across gender with increasing age, regardless of GFR estimating equation. Also the CG/BSA equation tended to overestimate CKD prevalence, unlike MDRD equation. There was a strong correlation between GFR estimates derived from two equations (*r *= 0.903, *p *< 0.001). The Bland-Altman limits of agreement were broad (Males = -4.27 to +45.95, Females = -11.88 to +28.82 ml/min/1.73 m^2^) and MDRD equation tended to overestimate eGFR when compared to CG equation (Figure 2).

**Table 1:** Prevalence of low eGFR and proteinuria in North India.

eGFR (ml/min/1.73 m^2^)	CG/BSA (*n *= 5252)	MDRD (*n *= 5252)
30–59	674 (12.8)	198 (3.8)

15–29	18 (0.3)	9 (0.2)

< 15	6 (0.1)	11 (0.2)

eGFR < 60 ml/min/1.73 m^2^	698 (13.3)	218 (4.2)

		

Proteinuria (≥ 1+): prevalence by eGFR strata		

30–59	42 (6.2)	27 (13.6)

15–29	9 (50)	5 (55.5)

< 15	6 (100)	10 (90.9)

eGFR < 60 ml/min/1.73 m^2^	57 (8.2)	42 (19.3)

		

Proteinuria (≥ 1+): prevalence in total population		

Proteinuria (≥ 1+)	118 (2.2)	118 (2.2)

Values are absolute numbers; numbers in parentheses are prevalence (in %). The prevalence estimates of low eGFR by CG/BSA equation were higher than MDRD equation. Proportion of subjects with proteinuria increased as eGFR decreased.

eGFR, estimated glomerular filtration rate; CG/BSA, Cockcroft-Gault equation corrected for body surface area; MDRD, modification of diet in renal disease equation.

### Prevalence of proteinuria

Semi quantitative assessment of proteinuria was done using visually read dipsticks. In the general population, 2.25% had proteinuria. There was an increasing prevalence of proteinuria with age, regardless of gender. Females aged above 60 years had a higher prevalence of proteinuria than males (5.76% vs. 3.46% respectively). Among subjects with proteinuria in the study population (*n *= 118/5,252), 35.6 to 48.3% had reduced eGFR depending on the GFR estimating equation used. Subjects with moderately reduced GFR (eGFR 30–59 ml/min/1.73 m^2^) formed a majority of subjects with low eGFR. In this subgroup only 6.2–13.6% had evidence of kidney damage in form of proteinuria. The proportion of subjects with proteinuria increased as eGFR decreased and almost all subjects with eGFR < 15 ml/min/1.73 m^2 ^had evidence of dipstick proteinuria (Table 1).

### Prevalence of major CKD risk factors

The study population had 31.2% prevalence of hypertension (HTN) and 7.3% prevalence of diabetes mellitus (DM). The prevalence of low eGFR in hypertensives was 17.1% as compared to 11.6% in non-hypertensives. Similarly, diabetics had a 19.3% prevalence of low eGFR as compared to 12.8% of non-diabetics. Prevalence of proteinuria was 3.4 times higher in diabetics than non-diabetics (1.9% vs. 6.5%) and 3.8 times higher in hypertensives than non-hypertensives (1.2% vs. 4.6%). The prevalence of obesity (BMI ≥ 25 kg/m^2^) was 26.8% and was comparable across gender.

### Characteristics of subjects with low eGFR

Subjects with renal impairment were compared with those without impairment, stratified according to gender. Renal impairment (eGFR < 60 ml/min/1.73 m^2^) was defined using CG/BSA equation for all further analysis. The results are tabulated in tables 2 and 3. The mean age of subjects with low eGFR was 54 years and 39.1% of those were aged 60 years or above. Males with renal impairment, as compared to those without, had higher SBP, DBP and fasting blood glucose levels. Subjects with low eGFR had higher waist circumference (WC) but lower BMI than their counterparts. Hemoglobin levels were also lower in the low eGFR group. There were a higher proportion of diabetics, hypertensives, proteinurics and subjects with low educational status and sedentary habits in the low eGFR group. Similar differences for two groups were found for females also, though there were no significant differences in hemoglobin levels and physical activity between the two groups. 28% of male subjects and 5.5% of females with renal impairment were current smokers, against 36.3% and 3% of those without. Similarly, 13.4% of males and 0.6% of females with low eGFR drank alcohol at least weekly, against 23% and 0.9% of those with normal eGFR. 34.8% of all low eGFR subjects gave family history of at least one amongst: kidney disease, HTN, DM, myocardial infarction (MI) or stroke. This was significantly higher than those without renal impairment (*p *< 0.001).

**Table 2:** Characteristics of males with and without renal impairment (eGFR_CG/BSA _< 60 ml/min/1.73 m^2^)

	eGFR < 60 ml/min/1.73 m^2^ (*n *= 350)	eGFR > 60 ml/min/1.73 m^2^ (*n *= 2805)	Crude OR	95% CI
Age (years)*	56.56 (13.52)	38.04 (11.77)		

Age above 60 years*	175 (50)	151 (5.4)	17.576	13.47–22.934

Education: Less than primary*	182 (52)	1203 (42.9)	1.443	1.155–1.802

Salaried job*	68 (19.4)	728 (26)	0.688	0.521–0.908

Current smoker*	98 (28)	1017 (36.3)	0.684	0.535–0.874

Current alcohol intake*	47 (13.4)	645 (23)	0.519	0.377–0.715

Exercise (≥ 60 minutes/day)	242 (69.1)	1985 (70.8)		

BMI (kg/m^2^)*	22.42 (3.44)	23.30 (3.69)		

Obese by BMI (≥ 25 kg/m^2^)*	70 (20)	757 (27)	0.676	0.514–0.890

WC (cm)*	83.84 (11.50)	81.12 (10.73)		

Obese by WC*	147 (42)	986 (35.2)	1.336	1.066–1.675

Systolic BP (mmHg)*	130 (87–230)	128 (80–200)		

Diastolic BP (mmHg)*	84 (57–150)	80 (55–138)		

Hypertension*	150 (42.9)	889 (31.7)	1.616	1.289–2.027

Proteinuria*	34 (9.7)	43 (1.5)	6.911	4.343–10.998

Serum creatinine (mg/dl)*	1.2 (0.67–6.30)	0.89 (0.40–1.40)		

eGFR CG/BSA* (ml/min/1.73 m^2^)	53.47 (10.71–59.95)	86.47 (60.02–207.54)		

Haemoglobin (g/dl)*	11.23 (1.34)	11.51 (1.31)		

Fasting blood glucose (mg/dl)*	98 (63–349)	92 (52–500)		

Diabetes mellitus*	47 (13.4)	210 (7.5)	1.917	1.367–2.687

NSAID intake*	41 (11.7)	194 (6.9)	1.786	1.250–2.552

Family history* CKD, HTN, DM, MI/Stroke	20, 72, 32, 16 (5.7, 20.6, 9.1, 4.6)	43, 261, 127, 37 (1.5, 9.3, 4.5, 1.3)	¶	¶

Total cholesterol (mg/dl)	171 (111–272)	171 (111–297)		

Values are absolute numbers, means or medians; numbers in parentheses are proportions, standard deviations or range.

* Differences or associations significant at *p *< 0.05.

¶ Odds ratio ranged from 2.5–3.5 with narrow confidence intervals (*p *< 0.05).

OR, odds ratio; 95% CI, 95% confidence interval; eGFR, estimated glomerular filtration rate; eGFR_CG/BSA_, eGFR by Cockcroft-Gault equation adjusted for body surface area; BMI, body mass index; WC, waist circumference; BP, blood pressure; NSAID, non steroidal anti inflammatory drugs; CKD, chronic kidney disease; HTN, hypertension; DM, diabetes mellitus; MI, myocardial infarction

**Table 3:** Characteristics of females with and without renal impairment (eGFR_CG/BSA _< 60 ml/min/1.73 m^2^)

	eGFR < 60 ml/min/1.73 m^2^ (*n *= 348)	eGFR > 60 ml/min/1.73 m^2^ (*n *= 1749)	Crude OR	95% CI
Age (years)*	51.47 (12.73)	34.26 (10.01)		

Age above 60 years*	98 (28.2)	42 (2.4)	15.932	10.84–23.416

Education: Less than primary*	282 (81)	1173 (67.1)	2.098	1.576–2.793

Salaried job*	14 (4)	155 (8.9)	0.431	0.246–0.754

Current smoker*	19 (5.5)	52 (3)	1.885	1.100–3.229

Current alcohol intake	2 (0.6)	15 (0.9)		

Exercise (≥ 60 minutes/day)	240 (69)	1252 (71.6)		

BMI (kg/m^2^)*	22.19 (3.41)	23.52 (4.28)		

Obese by BMI (≥ 25 kg/m^2^)*	64 (18.4)	515 (29.5)	0.537	0.402–0.718

WC (cm)*	78.36 (10.24)	77.10 (11.67)		

Obese by WC*	156 (44.8)	687 (39.3)	1.382	1.097–1.741

Systolic BP (mmHg)*	130 (100–200)	124 (85–240)		

Diastolic BP (mmHg)*	80 (60–114)	80 (50–150)		

Hypertension*	130 (37.4)	468 (26.8)	1.631	1.281–2.077

Proteinuria*	23 (6.6)	18 (1.1)	6.806	3.632–12.754

Serum creatinine (mg/dl)*	1.0 (0.6–6.20)	0.8 (0.4–1.30)		

eGFR CG/BSA* (ml/min/1.73 m^2^)	53.77 (8.69–59.99)	81.58 (60.06–186.08)		

Haemoglobin (g/dl)	10.79 (1.08)	10.88 (1.07)		

Fasting blood glucose (mg/dl)*	95 (55–412)	89 (52–558)		

Diabetes	27 (7.8)	99 (5.7)		

NSAID intake*	61 (17.5)	177 (10.1)	1.888	1.375–2.592

Family history* CKD, HTN, DM, MI/Stroke	5, 59, 31, 8 (1.4, 17, 8.9, 2.3)	13, 124, 69, 7 (0.7, 7.1, 3.9, 0.4)	¶	¶

Total cholesterol (mg/dl)	168.5 (111–289)	169 (112–312.90)		

Values are absolute numbers, means or medians; numbers in parentheses are proportions, standard deviations or range.

* Differences or associations significant at *p *< 0.05.

¶ Odds ratio ranged from 2.1–2.6 with narrow confidence intervals (*p *< 0.05).

OR, odds ratio; 95% CI, 95% confidence interval; eGFR, estimated glomerular filtration rate; eGFR_CG/BSA_, eGFR by Cockcroft-Gault equation adjusted for body surface area; BMI, body mass index; WC, waist circumference; BP, blood pressure; NSAID, non steroidal anti inflammatory drugs; CKD, chronic kidney disease; HTN, hypertension; DM, diabetes mellitus; MI, myocardial infarction

### Association between low eGFR and risk factors

In unadjusted univariate analysis for males (Table 2), presence of diabetes (OR; odds ratio 1.92, 95% confidence interval 1.37–2.69) and hypertension (OR 1.62, CI 1.29–2.03) associated with renal impairment. Low educational status, unsalaried job and non-steroidal anti-inflammatory drugs (NSAID) intake, independent of other covariates, also associated with presence of renal impairment. Males with family history of cardiac/renal risk factors had at least twice the odds of having low eGFR than those without such history. Central obesity (as assessed by WC) also associated with renal impairment (OR 1.34, CI 1.07–1.67). However, smoking and alcohol status associated inversely with low eGFR (OR_smoking _0.68, CI 0.53–0.87; OR_alcohol _0.52, CI 0.38–0.71). Males classified as obese by BMI had less odds of having renal impairment (OR 0.68, CI 0.51–0.89). Subjects with proteinuria had higher odds of having renal impairment (OR 6.91, CI 4.34–10.99)

Similar associations between renal impairment and covariates were found for females also. Hypertension (OR 1.63, CI 1.28–2.08), NSAID intake (OR 1.89, CI 1.37–2.59), low educational status (OR 2.1, CI 1.58–2.79), central obesity (OR 1.38, CI 1.10–1.74), presence of proteinuria (OR 6.81, CI 3.63–12.75) and family history of cardiac/renal risk factors all associated significantly with renal impairment in univariate analysis. Current smoking also associated with low eGFR (OR 1.88, CI 1.10–3.23) but alcohol intake did not associate significantly. However, unlike males, diabetes did not associate with renal impairment in unadjusted analysis. Like males, females with high BMI also had less odds of having renal impairment (OR 0.54, CI 0.40–0.72).

A multivariate logistic regression was done adjusting for all covariates and two-way interactions between variables. Variables not contributing significantly were excluded and model was refitted till all the variables contributed significantly to explain the probability of renal impairment. Analysis was done separately for CG and MDRD equation estimated GFR. The results are tabulated in table 4. The only significant associations of renal impairment were age above 60, female gender, low educational status, central obesity, NSAID intake, hypertension, diabetes and presence of proteinuria. The risk factor profiles obtained by CG and MDRD logistic regression models were almost similar.

**Table 4:** Association between selected risk factors and renal impairment in the survey population: Multivariate logistic regression.

	OR (95% CI) Model 1^#^	OR (95% CI) Model 2^#^
Age above 60 years	29.489 (21.417–40.604)	13.499 (8.908–20.456)

Gender (Females)	1.529 (1.090–2.145)	3.259 (2.399–4.426)

Education: Less than primary	1.309 (1.013–1.693)	*ns*

Obese by BMI	0.401 (0.310–0.517)	*ns*

Obese by WC	1.338 (1.084–1.652)	1.577 (1.168–2.129)

Hypertension	1.735 (1.388–2.169)	1.370 (1.024–1.833)

Diabetes mellitus	1.513 (1.048–2.184)	2.154 (1.289–3.598)

Proteinuria	16.728 (8.408–33.281)	14.645 (9.167–23.395)

NSAID intake	1.337 (1.010–1.770)	*ns*

Model 1 = Using Cockcroft-Gault equation estimated glomerular filtration rates.

Model 2 = Using Modification of Diet in Renal Disease equation estimated glomerular filtration rates.

# All associations significant at *p *< 0.05

OR, odds ratio; 95% CI, 95% confidence interval; BMI, body mass index; WC, waist circumference; NSAID, non steroidal anti inflammatory drugs; *ns*, not significant.

### Association between proteinuria and risk factors

Age above 60 years, NSAID intake, obesity (as defined by BMI), central obesity (as assessed by WC), hypertension and diabetes associated significantly with proteinuria in univariate analysis (Table 5). After adjusting for confounding by other covariates in multivariate logistic regression only age above 60 years, NSAID intake, central obesity, hypertension and diabetes effects remained significant. Approximately 1 in 10 subjects with diabetes and hypertension had proteinuria against 1 in 73 for subjects without diabetes and hypertension.

**Table 5:** Risk factors associated with proteinuria in multivariate logistic regression*.

	Unadjusted OR	95% CI	Adjusted OR	95% CI
Age group				
• 20–39	Referent	Referent	Referent	Referent
• 40–59	2.888	1.900–4.389	1.759	1.133–2.731
• Above 60	3.920	2.261–6.794	2.103	1.184–3.735

NSAID intake	3.611	2.359–5.527	2.385	1.532–3.712

Obese by BMI	2.120	1.465–3.068	*ns*	*ns*

Obese by WC	2.859	1.960–4.169	1.980	1.337–2.934

Hypertension	3.989	2.729–5.830	2.755	1.855–4.091

Diabetes mellitus	3.586	2.277–5.649	2.168	1.349–3.484

* All associations significant at *p *< 0.05. Proteinuria was defined as ≥ 1+ on dipstick.

OR, odds ratio; 95% CI, 95% confidence interval; BMI, body mass index; WC, waist circumference; NSAID, non steroidal anti inflammatory drugs; *ns*, not significant.

### Awareness of renal impairment

Only 3.3% of subjects with low eGFR were aware of their renal impairment. Majority of these had eGFR < 30 ml/min/1.73 m^2^. Females with renal impairment were less aware than males (1.4% vs. 5.1%).

## Discussion

This is the first community-based study to estimate and compare the prevalence of low glomerular filtration rate (low eGFR) in North Indian population using Cockcroft-Gault (CG) and Modification of Diet In Renal Disease (MDRD) equation. Our results suggest that burden of renal impairment may be substantial and there is a strikingly high prevalence of renal and cardiovascular disease risk factors in North Indian community. The period prevalence of renal impairment in our study population averaged from 4.2 to 13.3%, depending on the estimating equation. We are aware of only two community-based studies assessing prevalence of chronic kidney disease (CKD) in India. While one placed the prevalence around 0.79% [3], the other estimated it to be 1.39% [5]. The former study assessed CKD prevalence in an urban setting of South Delhi using a serum creatinine cutoff of > 1.8 mg% to define renal failure. The latter study assessed CKD prevalence primarily in a rural setting using MDRD equation. Both studies had their limitations. Estimates of CKD based on serum creatinine cutoffs are confounded by many covariates and are generally considered crude for epidemiological studies, besides causing significant underestimation of prevalence [16,17]. The other study though used MDRD equation, had a major limitation of being based in a rural population, which is known to have a significantly lower prevalence of CKD associated risk factors [18]. Also in that study only those subjects with urinary abnormalities or a positive response to a risk factor assessment questionnaire underwent blood testing, thus raising concerns of an ascertainment bias. Moreover, both population-based studies did not comment on distribution of CKD prevalence across gender and age groups.

Our study partly overcomes these limitations and gives an assessment of prevalence of low eGFR in North Indian community using eGFR cutoffs established by K/DOQI guidelines. We used both Cockcroft-Gault equation corrected for body surface area (CG/BSA) and Modification of Diet in Renal Disease (MDRD) equation for estimating prevalence of renal impairment in our study population. Though the validity of these equations to estimate GFR in Indian population has been questioned by few hospital based studies [6,7] there is no community-based study which compares the GFR estimates derived from these two equations. The use of serum creatinine based GFR estimating equations also ensured crude comparisons with prevalence data from other countries.

Using CG/BSA equation and a representative population; our study estimated the prevalence of low eGFR (eGFR < 60 ml/min/1.73 m^2^) to be 13.3%. With MDRD, the prevalence was estimated to 4.2%. These figures are comparable to studies from many other countries, including some western populations [8,19-23]. The mean age for renal impairment group in our study was 54 years, which is very similar to another community-based study in Delhi [3]. Significant impairment of renal function was seen with increasing age and was worse in females. This is consistent with studies assessing prevalence of CKD in other nations [21].

The difference between GFR estimates derived from CG/BSA and MDRD equation can be explained partly by their non-validation in Indian population and lack of an Indian subgroup in the original study populations from which these equations were derived. For instance, accurate estimation of GFR by MDRD equation requires coefficients specific for indigenous population [24]. However such coefficient or correction factor has not yet been derived/validated for Indian population. The overestimation of GFR by MDRD equation can also be explained by its inherent positive bias in Indian subjects [6]. In our study also MDRD equation had a positive bias and variance when compared to CG/BSA equation. Though there was a strong correlation between GFR estimates derived from two equations, the broad limits of agreement suggest that determination of true eGFR in a subject is highly dependent on the equation used.

Diabetes and hypertension are known to play an important role in CKD and not surprisingly diabetics (19.3% vs. 12.8%) and hypertensives (17.1% vs. 11.6%) had a higher prevalence of renal impairment than their healthy counterparts. Majority of subjects aged 60 years or above (62.9%) had diabetes or hypertension or both as compared to 36% of subjects below 60 years of age. This, coupled to physiological gradual decline in renal function with age, may explain the increase in prevalence of low eGFR with increasing age. These prevalence figures are comparable to few recent studies assessing prevalence of HTN and DM in Indian populations [25-28]. However only 20% of hypertensives and 57% of diabetics were aware of their diagnostic status.

Family history of kidney disease and cardiac/renal risk factors significantly associated with renal impairment. This underscores the influence of genetic factors in CKD. Studies evaluating risk of developing CKD in relatives of these "high risk" groups have supported the notion of clustering of CKD in families and few even recommend regular screening for CKD in such groups [29,30].

Another interesting observation was the association between renal impairment and low educational status. Many studies across nations [19,31] and also studies from India [32], have found similar associations. Multiple factors including higher prevalence of obesity and non-communicable disease risk factors, poor health seeking behavior and congenital factors like developmental endowment with fewer nephrons due to prematurity and low birth weight, have been suggested as possible explanations.

The inverse association between BMI and renal impairment seems paradoxical. Since BMI assesses the entire body mass including: central and peripheral fat, muscle mass and fluid, it is generally believed to be a less sensitive estimate of obesity in CKD patients [33]. Since it is the central obesity which determines the cardiovascular and renal risk, a measure that assesses visceral fat will be preferable in these subjects. Waist circumference (WC) and waist:hip ratio (WHR) are two such markers. These markers of central obesity have been shown to be better associated with of renal and cardiovascular risk than BMI in longitudinal and cross-sectional studies [34,35]. Indeed our study shows an association between undesirable WC and renal impairment. We preferred to use WC over WHR as WC is more sensitive index of upper body adiposity than WHR in Indian population [15].

Our study also suggests an inverse association between renal impairment and smoking and alcohol status. Similar observations were documented in a cross-sectional study in an Australian Aboriginal community [23]. It was suggested that in populations with low per capita income cigarette smoking marked relative affluence, which itself protected against renal disease. A cross-sectional study from Japan also suggested an inverse association between current smoking and low eGFR [36]. The possibility of a survivor bias in cross-sectional design can also explain this inverse association. It's likely that smokers and alcoholics die before their renal impairment can be detected from other causes like cardiovascular events or cirrhosis. Another possible explanation could be related to change in lifestyle among subjects aware of their renal impairment status. However, the low awareness of CKD in our study population makes this hypothesis less plausible. However, evidence on the contrary is also available in the literature. Some cross-sectional studies have demonstrated a positive association between smoking, alcohol intake and renal impairment [37,38]. This discrepancy in results could be related to certain unknown factors. Interestingly, the effects of smoking and alcohol were rendered insignificant when adjusted for other covariates in logistic regression, thus suggesting confounding by other variables.

The awareness regarding renal impairment in our study population was dismal. This probably reflects on poor perception of general public and practitioners regarding the magnitude of CKD in India. In unpublished observations by Ram Prabhakar *et al*, less than 20% of general practitioners could define CKD while less than 12% were aware of MDRD equation to estimate GFR [39]. This further emphasizes the need to increase awareness on CKD in general public as well as medical fraternity.

The prevalence of proteinuria was 2.25% in the study population. One cross-sectional study from a rural population in India estimated a lower prevalence (0.47%) [40] while another cross-sectional study from an urban locale estimated a higher prevalence (4.41%) [3]. Since our study encompassed all socioeconomic spectra, our prevalence estimates may be closer to the actual values. The presence of proteinuria significantly associated with low eGFR. There was an increasing prevalence of proteinuria as GFR decreased, thus corroborating proteinuria as a marker of kidney disease progression. This suggests that subjects with proteinuria should be worked up adequately for an underlying renal impairment as proteinuria is a significant mortality predictor [41].

Our study has few limitations. The major limitation lies in using GFR estimating equations not validated for Indian population. Though CG and MDRD are routinely used in clinical situations, the accuracy and reliability of these equations to predict GFR in Indian population is at best questionable. It is well recognized that Indian population has a lower normal range of GFR than western populations [7,42]. This is in part related to anthropometric phenotype, low protein intake and possible genetic endowment with fewer nephrons. Since these equations were developed from western cohorts, they are more likely to classify an Indian subject with normal GFR into renal impairment group. Besides how reliable these equations are in subjects with normal serum creatinine is also debated, independent of effects of ethnicity [17,43]. The application of these equations in epidemiological studies has also been questioned [44]. It will be interesting to assess CKD prevalence with a GFR estimating equation validated for Indian population. A new 'Apollo Chennai GFR equation' has been proposed but it needs validation [7]. Another limitation of our study is that we did not calibrate serum creatinine assays. It is generally accepted that calibrating serum creatinine assays improves performance of GFR predicting equations, particularly at higher values [45]. This also limited us from staging CKD using K/DOQI guidelines. The use of fasting blood glucose rather than oral glucose tolerance test may have led to an underestimation of true prevalence of diabetes mellitus. Similarly, using a single measure of dipstick proteinuria is likely to have caused an overestimation especially since no adjustment for urinary protein concentration was made. For instance, in a study population in United States, on a repeat measurement only 63% of subjects with proteinuria had a positive result [46]. Use of more specific measures like urine albumin:creatinine ratio (ACR) to assess micro- and overt albuminuria would be desirable. Finally, cross-sectional nature of our study does not allow one to derive a causal inference. Caution must be exercised in extrapolating this data to determine true community prevalence in view of these shortcomings.

## Conclusion

Our study highlights the emerging issue of CKD in India. The prevalence of low eGFR in India is similar to many other tropical nations and with epidemiological transition to lifestyle disorders, rising prevalence of HTN and DM and a substantial geriatric population, the situation has the potential to become a future public health problem. Increasingly it is being recognized that CKD is an additional cardiac risk factor and CKD patients are also at a higher risk of cardiovascular morbidity [47]. Considering the costs of CKD management, poor CKD awareness and deficit of trained nephrologists and other health care professionals, the only viable and cost effective option, at present, seems to be in primary prevention and screening, at least for "high risk" groups. We recommend screening efforts directed at females, elderly and people with low educational status. In addition, office dipstick detection of proteinuria can pick up CKD early. Since it is amenable to early intervention, significantly reducing mortality, subjects with proteinuria should be adequately worked up by the primary care provider. Regular follow up has been shown to benefit subjects with renal impairment [48]. In view of different racial, dietary and anthropometric profile of South Asians/Indians, studies validating GFR estimating equations derived from Western cohorts are needed. It will be desirable to have a serum creatinine based GFR estimating equation validated for South Asian population through large, multicentric, cohort studies.

## Competing interests

The authors declare that they have no competing interests.

## Authors' contributions

NPS and GKI conceived the study idea, design and reviewed the manuscript draft. AJ, PB, ML and GSM carried out the field activities. VKS drafted the manuscript and did the literature review. All authors read and approved the final manuscript.

## Pre-publication history

The pre-publication history for this paper can be accessed here:

http://www.biomedcentral.com/1471-2369/10/4/prepub
